# Defects and Defect Passivation in Perovskite Solar Cells

**DOI:** 10.3390/molecules29092104

**Published:** 2024-05-02

**Authors:** Zhanwei Wang, Hongli Gao, Dandan Wu, Junhua Meng, Jinxiang Deng, Min Cui

**Affiliations:** School of Physics and Optoelectronic Engineering, Beijing University of Technology, Beijing 100124, China

**Keywords:** defect passivation, perovskite solar cells, ionic compounds, organic molecules

## Abstract

Perovskite solar cells have made significant strides in recent years. However, there are still challenges in terms of photoelectric conversion efficiency and long-term stability associated with perovskite solar cells. The presence of defects in perovskite materials is one of the important influencing factors leading to subpar film quality. Adopting additives to passivate defects within perovskite materials is an effective approach. Therefore, we first discuss the types of defects that occur in perovskite materials and the mechanisms of their effect on performance. Then, several types of additives used in perovskite solar cells are discussed, including ionic compounds, organic molecules, polymers, etc. This review provides guidance for the future development of more sustainable and effective additives to improve the performance of solar cells.

## 1. Introduction 

Perovskite solar cells (PSCs), which are third-generation solar cells, have attracted widespread attention because of their remarkable ability to rapidly increase power conversion efficiency (PCE), increasing rapidly from an initial value of 3.8% to the currently certified 26.1% over the past decade [1]. This is mainly attributed to the excellent optoelectronic characteristics of perovskite materials, including tunable direct bandgaps [2], high absorption coefficients (≈10^5^ cm^−1^) [3,4], bipolar charge carrier transport [5,6], long carrier diffusion distance (>1 μm) [7,8], and high defect tolerance. Furthermore [9,10,11], compared to traditional silicon solar cells, perovskite solar cells are less expensive (GW-level costs can be only 3.5–4.9 US cents kWh^−1^ after industrialization) [12].

**Figure 1:**
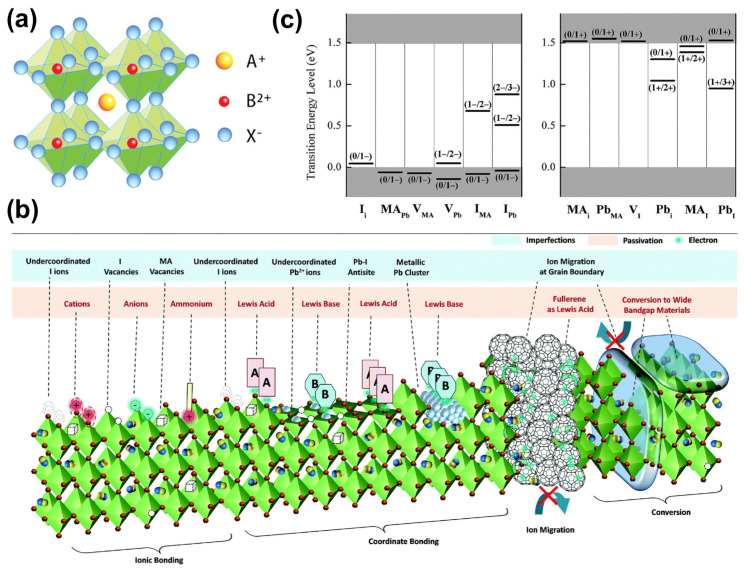
(**a**) Structural features of ABX_3_ perovskites. This material was reprinted with permission from Ref. [13] (copyright 2019, John Wiley and Sons). (**b**) Defect distribution in perovskite film. This material was reprinted with permission from Ref. [15] (copyright 2019, Royal Society of Chemistry). (**c**) The transition energy levels of (**left**) intrinsic acceptors and (**right**) intrinsic donors in MAPbI_3_. This material was reprinted with permission from Ref. [9] (copyright 2014, American Institute of Physics).

The light-absorbing layer in PSCs mainly consists of metal halide perovskites, with the general formula of ABX_3_. The A-site is typically occupied by organic molecules (usually methylammonium (MA^+^) or formamidinium (FA^+^)) or metal cations (mainly cesium ions (Cs^+^)), and the B-site is usually occupied by divalent Pb^2+^ or Sn^2+^, while the X-site is generally occupied by halide ions (I^−^, Br^−^, and Cl^−^). The crystal structure of perovskite is typically cubic or octahedral, as shown in Figure 1a [13]. The A ions are located at the center of the cubic unit cell, and the B ions are located at the corners of the unit cell, forming [BX_6_]^4−^ octahedra with six X halide ions. The stability of perovskite’s crystal structure is primarily determined by the corresponding tolerance factor (τ = (*R_A_* + *R_X_*)/$\sqrt{2}$·(*R_B_* + *R_X_*)) and octahedral factor (*μ* = *R_B_*/*R_X_*), where *R_A_*, *R_B_*, and *R_X_* represent the ionic radius of A, B, and X ions, respectively. When 0.81 < *τ* < 1.11 and 0.44 < *μ* < 0.90, ABX_3_ forms the perovskite structure. When *τ* = 1.0, it displays a cubic structure, with the highest symmetry. When *τ* is between 0.89 and 1.0, the lattice structure becomes rhombohedral (a trigonal system), transforming into an orthorhombic structure when *τ* < 0.96 [14]. 

The ideal crystal structure is formed by atoms in a periodic arrangement; however, in the actual preparation process, defects are inevitable in perovskite films due to the nature of the ionic crystals of perovskite and the rapid and uncontrollable crystallization process occurring at this time [15,16,17,18]. Three main types of defects can be distinguished in PSCs (Figure 1b) [15]: (1) zero-dimensional (0D) point defects, such as intrinsic defects (vacancies, interstitials, or antisite substitution defects) and foreign atoms (impurities or dopants (Frenkel defects and Schottky defects are the most common point defects [15]); (2) one-dimensional (1D) or two-dimensional (2D) defects [18], with one-dimensional defects mainly corresponding to dislocation defects formed by the local irregular arrangement of atoms, while perovskite grain boundaries and surfaces with dangling bonds are two-dimensional defects, and the surfaces and grain boundaries of perovskite films are where these defects are mainly found, not only creating severe non-radiative composites but also providing pathways for oxygen penetration, adversely affecting the performance and long-term stability of PSC devices; and (3) three-dimensional (3D) defects [19], mainly corresponding to agglomerates, pinholes, etc.

The presence of defects introduces defect energy levels into the energy level structure of perovskite, which can be categorized into deep-energy-level defects and shallow-energy-level defects according to the position of the defect energy level from the valence band maximum (VBM) or conduction band minimum (CBM) [20,21]. As an example, the typical point-space defect energy level of MAPbI_3_ perovskite is located in the energy band (Figure 1c) [9]. Shallow energy level defects are farther away from the band gap center and closer to the top of the valence band or the bottom of the conduction band [15]. Therefore, the trapped carriers are likely to be re-excited into the valence band or conduction band to participate in the transport, and the shallow-energy-level trap has almost no effect on the carrier complex. While the deep-energy-level defects are closer to the forbidden band’s center, the trapped carriers have difficulty becoming excited into the conduction band or valence band again and are often consumed by non-radiative composites, thus seriously affecting the performance of PSC devices [22,23]. The deep-energy-level defects are mainly located at grain boundaries and interfaces with high formation energies. Perovskite films prepared using solution methods are usually polycrystalline with many grain boundaries. Results have shown that the defect density in polycrystalline perovskite films is about 10^16^–10^17^ cm^3^, while that in single-crystal perovskite is only 10^9^–10^10^ cm^3^ [24]. Most of the defects in polycrystalline perovskite are distributed in grain boundaries or interfaces. Therefore, the effective repair of defects at grain boundaries or interfaces can significantly enhance the performance of PSCs [25]. 

In addition, a lack of long-term stability and the toxicity of lead currently hinder the commercialization of perovskite. The decomposition of the perovskite materials is the main factor of instability [15]. On the one hand, perovskite materials are inherently unstable. On the other hand, perovskite materials are susceptible to accelerated decomposition in water, oxygen, light, heat, and other media. At the same time, the stability of q solar cell is also affected by the interface defects between the electron/hole transport layer and the perovskite layer. Moreover, the atoms/ions from the metal electrode can easily penetrate into the perovskite and react with it, reducing the stability of a device. Furthermore, the soluble lead in perovskite is detrimental to the long-term application of PSCs, and this factor is harmful to the environment and human health. Strategies such as chemical absorption or physical encapsulation are commonly used to effectively mitigate the problem of lead leakage [26]. With chemical absorption, lead leakage can be reduced by complexing lead ions with additives, such as sulfonic-acid-based lead-adsorbing resin, phosphates, and titanium dioxide [26,27,28].

Various methods have been employed in perovskite solar cells to effectively passivate defects and improve efficiency [15,18,29,30]. These methods include interface engineering [31], additive engineering [32], molecular design [33], and composition regulation [34]. Among these techniques, additive engineering has been widely applied, yielding remarkable results in experiments. In this review, we provide a systematic introduction to defect passivation in perovskite solar cells, including the effect of defects on devices, and the influence of different types of additives on the PCE of perovskite solar cells. This work will offer relevant guidance for the design and enhancement of PCE through the utilization of additives.

## 2. Passivation of Defects in Perovskite Films

Perovskite film is the core functional layer. The quality of this film directly determines the efficiency and stability of PSCs. However, there are a lot of defects in perovskite films, and they have a serious impact on the PCE of solar cells. Therefore, employing a series of passivating agents to passivate defects in the perovskite layer is crucial for obtaining high-quality films, offering high crystallinity, low defect density, dense morphology, and high charge carrier mobility, which are beneficial for attaining excellent PCE and stability with respect to perovskite solar cells.

### 2.1. Ionic Compounds

The main defects in perovskite films are point defects, including insertion and substitution defects with high formation energies as well as vacancy defects with low formation energies. Due to the ionic crystal nature of perovskite films, coordination compounds with charged ions are used to introduce and form ion bonds with defect sites for defect passivation. 

#### 2.1.1. Cations

**Figure 2:**
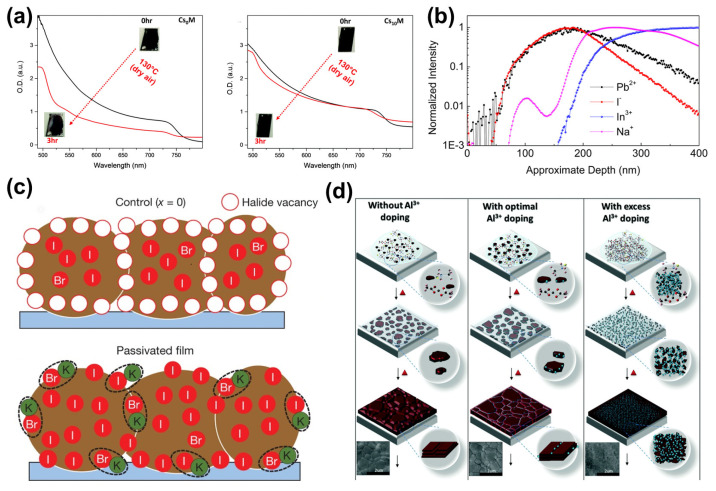
Passivation by cations. (**a**) UV-visible absorption of Cs_0_M and Cs_10_M films annealed at 130 °C for 3 h in dry air with the corresponding images. This material was reprinted with permission from Ref. [37] (copyright 2016, Royal Society of Chemistry). (**b**) SIMS measurement of MAPbI_3_ films grown on an ITO glass substrate. This material was reprinted with permission from Ref. [39] (copyright 2017, American Chemical Society). (**c**) Schematic of a cross-section of a film showing halide-vacancy management in cases of excess halide, in which the surplus halide is immobilized through complexation with potassium into benign compounds at the grain boundaries and surfaces. This material was reprinted with permission from Ref. [40] (copyright 2018, Springer Nature). (**d**) Schematic diagram of the proposed perovskite polycrystalline thin-film growth and influence of Al^3+^. This material was reprinted with permission from Ref. [41] (copyright 2016, Royal Society of Chemistry).

In organic–inorganic hybrid perovskite solar cells, the organic amine cations in the perovskites are volatile and can easily evaporate during high-temperature annealing, leading to vacancy defects. Ion doping is an effective method of passivating vacancy defects. Ions with appropriate radii can be adopted to replace A-site ions in perovskites. Among them, cesium ions (~167 pm) and rubidium ions (~152 pm) are the most representative [35,36]. For example, methylammonium ions in the lattice of MAPbI_3_ perovskites can be replaced by cesium ions, forming mixed A-site ions with a more stable crystal phase in Cs_x_MA_1−x_PbI_3_ perovskites. In 2016, Saliba et al. found that Cs^+^ can suppress the yellow phase of perovskites, making perovskite crystallization more stable and less susceptible to environmental influences (Figure 2a) [37]. In the same year, this team introduced Rb^+^ into the active perovskite phase, as Rb can stabilize the black phase of FA perovskite and be integrated into PSCs. The resulting perovskite solar cell yielded a V_oc_ of 1186mV [33]. Similarly, in inorganic perovskite solar cells, Guo et al. doped RbI into the CsPbI_2_Br precursor: the resulting films had excellent crystallinity, with a surface morphology free of pinholes [38].

Some alkali metal ions, such as potassium, sodium, and lithium cations, have also been studied for defect passivation [39,40,42,43,44]. In 2017, Bi et al. found that Na^+^ in a substrate can diffuse into the perovskite layer in a device to enhance its PCE by passivating the grain boundaries in the perovskite (Figure 2b) [39]. Subsequently, Abdi-Jalebi et al. systematically investigated the passivation mechanism of KI in perovskite solar cells [40]. It has been shown that K^+^ ions can combine with uncoordinated halide ions to passivate the vacancy defects of A-site cations, with other K^+^ ions accumulating at the grain boundaries to inhibit ion migration (Figure 2c). Son et al. further showed that K^+^ was able to enter the perovskite lattice, occupying the sites of possible insertion defects and preventing the formation of insertion defects. At the same time, they also showed that potassium ions were able to prevent the formation of Frenkel defects, so K^+^ was also able to eliminate hysteresis, and the V_oc_ of forward scanning was almost the same as the reverse scanning curve after the addition of KI [42]. 

Divalent or trivalent metal cations are also used regularly as passivators. Wang et al. introduced aluminum acetylacetonate (Al-acac_3_) into a perovskite precursor solution and found that the presence of Al^3+^ can reduce strain in polycrystalline films (Figure 2d) [41]. Perovskite crystals with better orientation and lower defect density were produced, resulting in an efficiency of up to 19.1% for inverted PSCs. Liu et al. introduced a small amount of Ni^2+^ into perovskite films to reduce the vacancy defects of Pb and I, resulting in an increase in PCE by 20.63% due to the high-quality films with large grain sizes [45]. Due to the oxidation of I^−^ to I^0^ and the reduction of Pb^2+^ to Pb^0^ during the manufacturing of perovskite devices, defect levels are introduced into the bandgap, causing non-radiative recombination, which is detrimental to the PCE and stability of the devices. Wang et al. doped rare-earth elements into perovskite films and found that the doped Eu formed Eu^2+^/Eu^3+^ ion pairs that oxidized Pb^0^ and eliminated I^0^ defects, achieving a high PCE of 21.52% and improving the stability of the corresponding device. Even after simulating continuous sunlight irradiation for 1500 h, the initial efficiency could still be maintained at 92% [46]. 

#### 2.1.2. Anions

**Figure 3:**
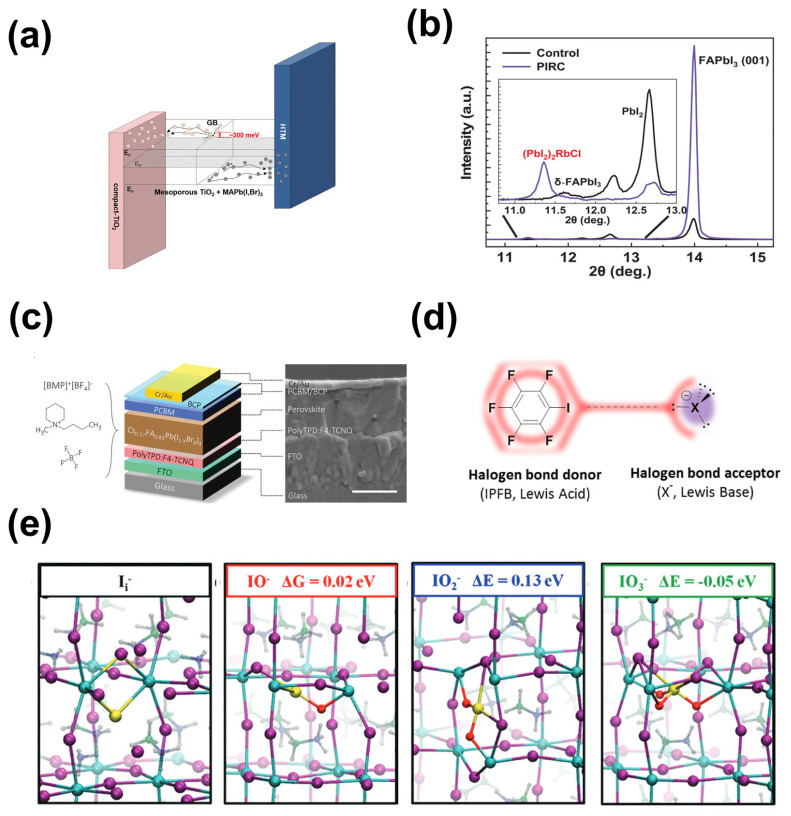
Passivation by anions. (**a**) A schematic band diagram near the GBs in Br-containing MAPb(I_0.88_,Br_0.12_)_3_ thin films; the charged GBs have a high localized built-in potential that improves carrier separation. This material was reprinted with permission from Ref. [54] (copyright 2015, American Chemical Society). (**b**) XRD of perovskite without RbCl and with 5% RbCl. This material was reprinted with permission from Ref. [56] (copyright 2022, The American Association for the Advancement of Science). (**c**) Schematic of the p-i-n perovskite solar cell and the chemical structure of [BMP]^+^[BF_4_]^−^. This material was reprinted with permission from Ref. [57] (copyright 2020, The American Association for the Advancement of Science). (**d**) Schematic diagram of the halogen bond interaction between halogen bond donor IPFB and halogen bond acceptor halogen anion, with sp^3^ hybridized valence electron. This material was reprinted with permission from Ref. [58] (copyright 2014, American Chemical Society). (**e**) Structure of I_i_^−^ and of its interaction products with 1/2 O_2_, O_2_, and 3/2 O_2_ along with the calculated energetics(ΔE, eV). Atom color code: yellow, I_i_; purple, I; red, O; light blue, Pb. Methylammonium cations are shadowed in the background for clarity. This material was reprinted with permission from Ref. [59] (copyright 2017, American Chemical Society).

Unlike cations, anions introduced into perovskite film are typically used to passivate defects with positive charges, such as Pb vacancies and halide vacancies. The research on anions mainly focuses on halide ions [47,48,49,50,51,52]. Studies have shown that adding an excess of iodide or iodine to perovskite precursor solutions can inhibit the formation of halide vacancies [53]. Similarly, due to the similar radii of bromide ions (~196 pm) and iodide ions (~220 pm), iodide ions in octahedral [PbI_6_]^4−^ can be replaced by bromide ions to adjust the bandgap of perovskite. Kim et al. reported that barrier bending occurred and defects at grain boundaries were passivated after introducing bromide into perovskites, suggesting that bromide ions promote the separation of electrons and holes at grain boundaries (Figure 3a) [54]. Moreover, the defects at grain boundaries or surfaces of perovskite films can be passivated by additives, such as PbCl_2_ [49]. Although some reports showed that Cl^−^ volatilized during annealing, some Cl^−^ ions still remained in the MAPbI_3_ film [49,55]. It should be noted that excess PbI_2_ can passivate defects at perovskite grain boundaries, but it may damage the stability of the device meanwhile. Zhao et al. used rubidium chloride (RbCl) to convert excess PbI_2_ into inactive (PbI_2_)_2_RbCI complexes and found that perovskite films were modified by this complex (Figure 3b), achieving a certified ultra-high PCE of 25.6% [56].

F, as the smallest atom in the halogen group with a radius of only 147 pm, has strong electronegativity [18]. Li et al. demonstrated that F^−^ plays a more significant role in passivating halide anion and organic anion vacancies compared to Cl^−^ and Br^−^ through experimental characterization and theoretical analysis [60]. This is because strong ionic bonds can be formed between F^−^ and Pb^2+^ and because hydrogen bonds (N-H····F) can form between F^−^ and organic cations (FA^+^/MA^+^) in perovskite film. Additionally, fluorides or fluorine-containing molecules typically exhibit excellent moisture-resistant and hydrophobic properties, so the stability of a device can be effectively enhanced by introducing fluoride-containing ionic liquids into the perovskite layer [57,61]. Lin et al. introduced a [BMP]^+^[BF_4_]^−^ ionic liquid (Figure 3c) into the perovskite layer and found that deep defects in the perovskite film were suppressed, thereby improving the V_oc_ and stability of the device [57]. Similarly, Bai et al. introduced the fluoride-containing ionic liquid-(BMIMBF_4_) into the perovskite layer; the BF_4_^−^ ion could improve the photovoltaic properties and stability of the film [61]. Abate et al. also used iodopentafluorobenzene to illustrate that fluoride bonding complexes can passivate halide ion defects (Figure 3d) [58].

In addition to halide anions, other anions can also serve as passivating agents [62,63,64]. For example, pseudo-halide ions like SCN^−^ can reduce recombination at grain boundaries through intermediate reactions [63]. Anionic carboxyl groups (-COO^−^) have also been widely used before. Jeong et al. added formate ions (HCOO^−^) to a perovskite precursor solution; this inhibited anionic vacancies at grain boundaries and the surface of the perovskite film, enhancing the film’s crystallinity and achieving a PCE exceeding 25% [65]. It should be noted that the passivation mechanism of carboxyl groups (-COOH) is different from that of (-COO^−^). -COO^−^ can passivate defects with positive charges through ionic interactions, while -COOH passivates negatively charged under-coordinated halides through forming hydrogen bonds [66]. Passivation strategies for -COOH will be discussed in Section 2.2.3. 

Moreover, studies have shown that water and oxygen can passivate the surfaces of perovskite films. D. Meggliolaro et al. proposed that oxygen can form oxide complexes, which could passivate deep hole defects via combination with iodide interstitial sites (Figure 3e) [59]. Yin et al. similarly found that O_2_^−^ has a passivating effect on the defects at grain boundaries [48].

### 2.2. Organic Molecules

In addition to passivating defects with ionic compounds, organic molecules containing specific functional groups are also excellent candidates for improving the film quality and enhancing the PCE and stability of these devices. Due to their larger molecular sizes, organic molecules generally cannot penetrate the interior of the perovskite lattice. However, the specific functional groups carried by organic molecules will interact with the ions located on the surface of perovskite film, effectively regulating the perovskite crystallization process and simultaneously repairing and passivating defects existing in the perovskite layer [67,68,69].

#### 2.2.1. Organic Ammonium Salt

**Figure 4:**
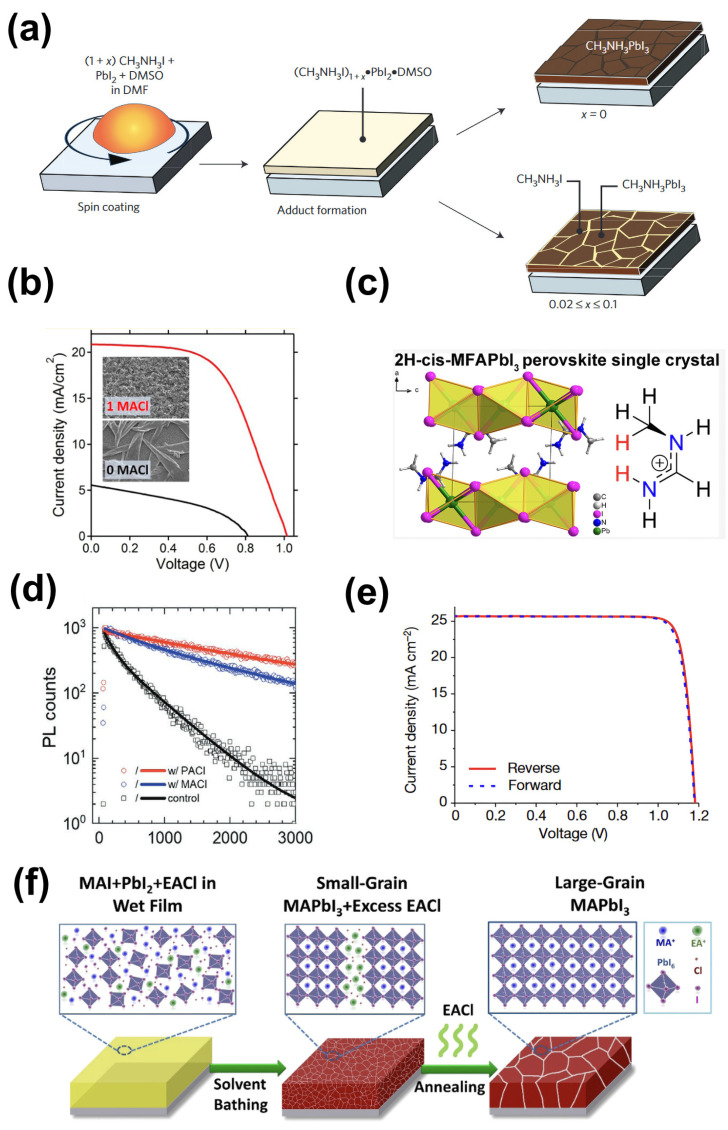
Passivation by alkylammonium halides. (**a**) Schematic of the coating process used to form CH_3_NH_3_PbI_3_ films with and without excess MAI. This material was reprinted with permission from Ref. [70] (Copyright 2016, Springer Nature). (**b**) *J*-*V* characterization of devices with and without MACI, with SEM scans of the films shown in the insets. This material was reprinted with permission from Ref. [72] (Copyright 2014, American Chemical Society). (**c**) Crystal structure of yellow MFAPbI_3_ single crystals with cis-MFA cations. This material was reprinted with permission from Ref. [74] (Copyright 2023, American Chemical Society). (**d**) TRPL of the perovskite films on glass substrate without (control) and with additive (MACl or PACl). This material was reprinted with permission from Ref. [34] (Copyright 2021, John Wiley and Sons). (**e**) *J*-*V* curves of the best-performing PSC fabricated with the target, which was measured in the reverse and forward modes under AM 1.5 G. This material was reprinted with permission from Ref. [32] (Copyright 2023, Springer Nature). (**f**) Schematic representation of EACl-MAPbI_3_ perovskite films nucleated/crystallized from a solvent bath followed by grain growth under thermal annealing in air. This material was reprinted with permission from Ref. [75] (Copyright 2018, Elsevier).

As a constituent of perovskite materials, organic ammonium salts can also induce defect passivation [32,34,70,71,72]. Son et al. introduced excess MAI into a perovskite precursor solution (Figure 4a) and found that dangling bonds at grain boundaries were passivated by excess MAI and thus suppressed non-radiative recombination, achieving a PCE of 20.1% and significantly reducing hysteresis in devices [70]. Regarding concentration at grain boundaries and passivation, Hawash et al. studied the influence of excess MAI at the interface between the perovskite layer and the hole transport layer, and the results indicated that the energy level adjustment is not in the MAI layer but in the dissociated species (the MAI layer dissociates on contact with methylammonium lead iodide) [71]. In addition, some studies have shown that materials containing excess MA in the presence of H_2_O can trigger anti-degradation reactions, delaying moisture degradation [73].

As a good additive, MACl has also been widely studied to improve the quality of perovskite films. Zhao et al. first introduced MACl into the process of preparing perovskite films, and the results showed that the crystallization process of MAPbI_3_ was effectively alleviated, which improved the coverage of the film on a planar substrate and enhanced the light absorption of the film (Figure 4b) [72]. Subsequently, MACl has been adopted in perovskite films in various systems to improve the performance of devices. Wang et al. first added MACl to the preparation process for FAPbI_3_ film and found that intermediate phases were induced during the growth of FAPbI_3_ crystals and then transformed into the α phase during thermal annealing, inhibiting the direct formation of the δ phase [76]. Kim et al. studied the mechanism of MACl in FAPbI_3_ films through theory and experiments, and their research indicates that the interaction between FA^+^ and I^−^ was enhanced by Cl, increasing the stability of the solar cells [77]. Meanwhile, the volume of MA^+^ was smaller than that of FA^+^, and the dipole moment of MA^+^ was greater than that of FA^+^, so a contraction of the crystal structure occurred due to the presence of MA^+^ in FAPbI_3_, further enhancing the stability of the perovskite crystal structure by reducing the volume of the perovskite cubic octahedron. Chen et al. found that intermediates named cis/trans-N-methyl iodomethylamine (MFAI) were produced by the reaction of FA^+^ and MA^0^, and the product could react with PbI_2_, producing the MFAPbI_3_ 2H-phase perovskite (Figure 4c) [74]. Jiang et al. introduced MACl into the precursor solution of organic salts and prepared (FAPbI_3_)_1−x_(MAPbBr_3_)_x_ orthorhombic perovskite using a two-step method, effectively improving the crystallization of perovskite films and achieving a PCE of over 21% for the prepared devices [78].

Next, we will discuss the influence of other alkylammonium chlorides (such as RACl) on perovskite films. In addition to propylammonium chloride (PACl), ethylammonium chloride (EACl) enhances film quality by passivating the defects at the grain boundaries of the film or affecting its phase transition [34,75]. Zhang et al. replaced MACl with long-chain propylammonium chloride (PACl), and the defects at grain boundaries were effectively passivated, with larger grains obtained [34]. As a result, carrier lifetime was significantly enhanced, increasing from 405 ns to 2110 ns (Figure 4d). Furthermore, the crystallization process of perovskite can be modulated by using combinations of different alkylammonium chlorides. Park et al. studied the effect of combinations of different RACl on the phase transition of perovskite films using grazing incidence wide-angle X-ray diffraction and scanning electron microscopy [32]. Their research indicated that the type and content of RACl were the key factors determining the phase transition. Adding certain amounts of PACl and MACl into the perovskite precursor made it easy to obtain high-quality α phase films, and the PCE of the devices increased by up to 25.73% (certified), which re-set the record for the highest efficiency (Figure 4e). Chloride salts can also improve film quality by passivating grain boundary defects without forming intermediate phases. Zhang et al. introduced ethylammonium chloride (EACl) into the perovskite precursor, resulting in micrometer-sized MAPbI_3_ films, which reduced defects at grain boundaries and surfaces without forming an intermediate that would affect the bandgap (Figure 4f) [75].

**Figure 5:**
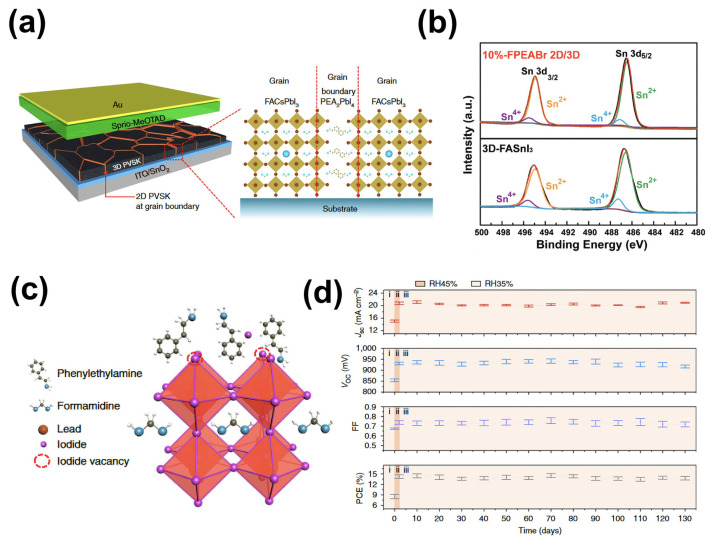
Passivation by organic ammonium salt. (**a**) Schematic of a device incorporating polycrystalline 3D perovskite film with 2D perovskite at grain boundaries. This material was reprinted with permission from Ref. [79] (copyright 2018, Springer Nature). (**b**) The X-ray photoelectron spectroscopy (XPS) spectra of Sn 3d in the FASnI_3_ and 10%-FPEABr films. This material was reprinted with permission from Ref. [85] (copyright 2021, John Wiley and Sons). (**c**) Possible passivation mechanism of the PEAI layer of perovskite film. This material was reprinted with permission from Ref. [86] (copyright 2019, Springer Nature). (**d**) Variations in J_sc_, V_oc_, FF, and PCE of devices upon fabricating and aging under ambient conditions without encapsulation: ((i) device fabrication without ambient exposure, RH35%; (ii) ambient exposure, RH45%; (iii) long-term storage, RH35%. This material was reprinted with permission from Ref. [87] (copyright 2017, Springer Nature).

The use of organic ammonium salts to form low-dimensional perovskite structures to passivate defects at grain boundaries (uncoordinated Pb^2+^, lead clusters, halide ion vacancies, etc.) can be applied to the perovskite precursor solution or on the perovskite surface [79,80,81]. Short-chain alkylammonium halides have been widely studied, including ethylammonium (EA), butylammonium (BA), iso-butylammonium (iso-BA), and phenethylammonium (PEA) [82,83,84]. The incorporation of a butylammonium cation (BA) into perovskite formed a layered 2D phase between 3D perovskite grains, whose heterogeneous structures reduced the density of defective states at the perovskite interface and thus suppressed non-radiative composites [83]. Lee et al. introduced phenethylammonium iodide (PEAI) into the FAPbI_3_ system, and the experimental results obtained showed that the formation of 2D perovskite PEA_2_PbI_4_ spontaneously aggregated at grain boundaries, significantly enhancing device stability (Figure 5a) [79]. Yu et al. replaced FAI with 4-fluorophenethylammonium bromide (FPEABr) to prepare tin-based perovskite, finding that the introduction of the 2D phase promoted highly oriented growth of FASnI_3_ [85]. Additionally, 2D perovskites can inhibit the oxidation of Sn^2+^ ions located at grain boundaries and on surfaces (Figure 5b).

Additionally, PEAI molecules can also be used to passivate surface defects in perovskites. Jiang et al. utilized PEAI to form a passivation layer on the surfaces of perovskite films, passivating lead and iodine defects at the grain boundaries and surfaces of the perovskite films [86]. Their study showed that the amine groups in PEAI molecules formed hydrogen bonds with Pb^2+^ and thus passivated defects, while iodine ions filled iodine vacancies (Figure 5c), thereby suppressing the non-radiative recombination of charge carriers and improving charge carrier transport. As a result, the devices achieved a PCE of 23.32%. Jiang et al. further used 4-fluorophenethylammonium (F-PEAI)iodide to passivate surface defects in perovskites [88]. It has been shown that the formation of iodine vacancies was significantly inhibited by F-PEAI, and the hydrophobicity of the film was simultaneously improved by the addition of fluorine, greatly enhancing the environmental stability of the devices. Other ammonium salts have also been used to improve the morphology and crystallinity of perovskite films for the preparation of high-performance PSCs. Among them, NH_4_Cl led to a significant enhancement in the crystallization of MAPbI_3_.

Additives based on ammonium salt derivatives (NH_4_Cl) could also reduce perovskite surface defects, improving film morphology and crystallinity [87,89,90]. Zuo et al. added NH_4_Cl additives to perovskite layers to prepare perovskite films with high crystallinity and a smooth uniform morphology, achieving a fill factor of 80.11% [89]. Furthermore, Rong et al. also utilized an NH_4_Cl additive to prepare high-quality MAPbI_3_ films under ambient conditions. During perovskite crystal growth, intermediate products (CH_3_NH_3_)_X_·NH_4_PbX_3_H_2_O_2_ (X = I or Cl) formed, assisting crystallization, and then the intermediate phase transformed into the perovskite phase during thermal annealing in the presence of water vapor in the air, promoting perovskite crystallization [87]. As a result, devices prepared with NH_4_Cl exhibited excellent stability, maintaining 96.7% of the initial PCE after being stored in an environment containing 35% RH for 130 days (Figure 5d).

#### 2.2.2. Lewis Acid

There are many electron-rich defects in perovskite films, such as under-coordinated halides and Pb-I anti-site defects, which can be regarded as Lewis bases [29,30,91]. By introducing an electron-withdrawing Lewis acid, Lewis acid-base adducts are formed by the interaction between Lewis acid and Lewis base defects. As a result, the defects will be passivated, and non-radiative recombination will be reduced, improving the photovoltaic performance of devices. Currently, fullerenes and their derivatives are the most widely used Lewis acids for passivating electron-rich defects [92,93,94,95].

**Figure 6:**
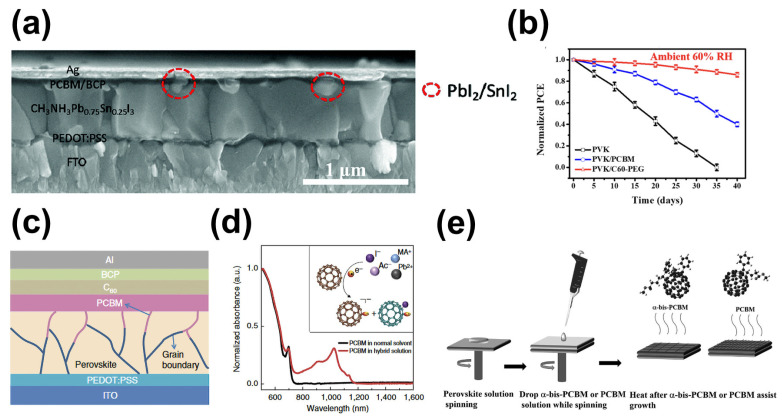
Passivation by Lewis acid. (**a**) Cross-sectional SEM image of inverted structure MAPb_0.75_Sn_0.25_I_3_ perovskite solar cell with C_60_ additive. This material was reprinted with permission from Ref. [96] (copyright 2017, Royal Society of Chemistry). (**b**) Standardized PCE and time stability testing of different PVCs (PVK, PVK/PCBM, and PVK/C_60_-PEG) stored under ambient conditions with RH 60% without encapsulation. This material was reprinted with permission from Ref. [91] (copyright 2019, American Chemical Society). (**c**) Device structure with PCBM layer; here, PCBM penetrated perovskite along grain boundaries. This material was reprinted with permission from Ref. [95]. Copyright 2014 Springer Nature). (**d**) Ultraviolet-visible absorption spectroscopy of the hybrid solution shows the interaction between PCBM and perovskite ions. This material was reprinted with permission from Ref. [98] (copyright 2015, Springer Nature). (**e**) Schematic diagram of the α-bis-PCBM- or PCBM-containing perovskite process. This material was reprinted with permission from Ref. [93] (copyright 2017, John Wiley and Sons).

Fullerene materials are favorable for carrier transport due to their high electron mobility and have attracted extensive attention as electron transport layers of perovskite solar cells. In fact, they are also good candidate materials for passivating defects. Liu et al. introduced C_60_ into MAPb_0.75_Sn_0.25_I_3_ films, passivating the defects both at the grain boundaries and surfaces in situ of perovskite films, successfully reducing pinholes in the film (Figure 6a) [96]. Furthermore, the development of novel fullerenes with specific functions can further promote the PCE improvement of PSCs. Fu et al. added fullerene-terminated polyethylene glycol (C_60_-PEG) into an anti-solvent to prepare perovskite films, increasing crystal size and passivating film defects (Pb-I defects and uncoordinated Pb atoms), resulting in an excellent PCE of 17.71% [91]. Additionally, C_60_-PEG significantly enhanced the moisture resistance of perovskite films, and the PCE of unencapsulated devices could maintain 93% of the initial value in a nitrogen atmosphere (25 °C; 60%RH) (Figure 6b). Liu et al. formed a heterojunction between fluorinated fullerene (DF-C_60_) and perovskite, and their results showed the defects both in perovskite crystals and at grain boundaries were effectively passivated by DF-C_60_, improving the charge collection and transport processes of the device and greatly reducing device hysteresis [97].

In addition to C_60_, phenyl-C_61_-butyric acid methyl ester (PCBM) has also been widely studied in relation to PSCs [95,99]. Shao et al. found that after spin-coating PCBM on the surface of perovskite, some PCBM can penetrate along the perovskite grain boundaries into the inner of perovskite (Figure 6c), thereby passivating defects in the grain boundaries and surfaces [95]. As a result, the defect density in the perovskite film treated with PCBM was reduced by two orders of magnitude. Using theoretical calculations and experimental characterization, Xu et al. found that PCBM reduced the hysteresis of the tested device and improved performance by passivating Pb-I anti-site defects in perovskite (Figure 6d) [98]. Chiang et al. demonstrated that PCBM effectively filled the pinholes and voids between perovskite grains, which improved the fill factor by up to 82%, and hysteresis was nearly eliminated [100]. Wang et al. achieved in situ gradient passivation of perovskite via spin-coating a mixture of PbI_2_ and PCBM as a pre-coating film before spin-coating the perovskite film, promoting electron extraction at the perovskite interface, reducing carrier non-radiative recombination, and resulting in a PCE of 20.1% [99].

Other fullerene derivatives can also passivate defects in perovskite films and improve the photovoltaic performance of devices [93,101,102]. Zhang et al. introduced α-bis-PCBM into perovskite films via an antisolvent method (Figure 6e) [93]. The vacancies and grain boundaries of perovskite films can be better filled by α-bis-PCBM, which enhances the crystallization of perovskite and accelerates electron extraction. α-bis-PCBM can also passivate the voids and pinholes in the hole transport layer, which enhances the stability of a device. Unsealed devices lose less than 10% PCE after 44 days of storage in air (65 °C; 40% RH). Li et al. introduced a cross-linkable [6,6]-phenyl C_61_-butyric acid styryl dendrimer (PCBSD) into perovskite layers in order to enhance electron extraction efficiency through the vacancies or pinholes created in the passivated bulk perovskite layers [102]. Vidal et al. showed that voids/pinholes and/or deep-narrow slits in perovskite films were passivated by C_70_ fullerene isomers, with the PCE improving from 19.3% to 20.5% [103].

#### 2.2.3. Lewis Base 

**Figure 7:**
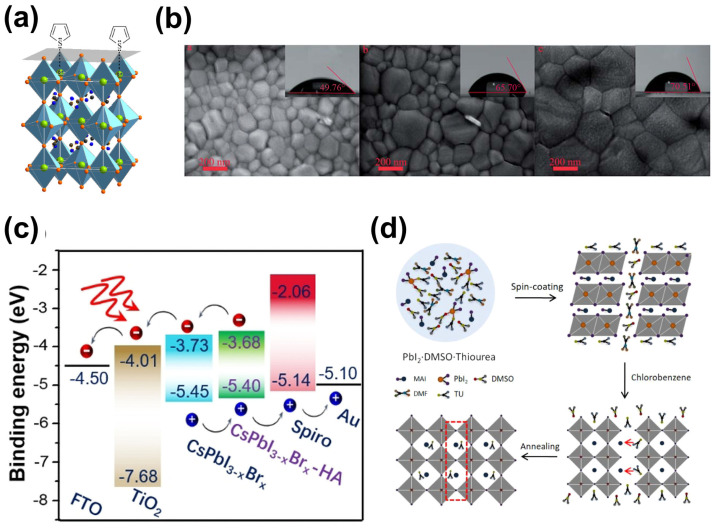
Passivation by Lewis acid with N and S functional groups. (**a**) Thiophene or pyridine molecules form ligand covalent bonds with Pb. Atom color code: cyan, Pb; orange, I; black, C; blue, N. This material was reprinted with permission from Ref. [107] (copyright 2014, American Chemical Society). (**b**) Top-view scanning electron microscopy (SEM) images of perovskite films containing no additive and those with 0.6 mg mL^−1^ of TDZT- and 0.6 mg mL^−1^ of TDZDT. This material was reprinted with permission from Ref. [109] (copyright 2018, Royal Society of Chemistry). (**c**) Energy-level diagram of PSCs passivated by HA. This material was reprinted with permission from Ref. [110] (copyright 2021, John Wiley and Sons). (**d**) Schematic diagram and schematic reaction process of the deposition of perovskite films. This material was reprinted with permission from Ref. [111] (copyright 2018, John Wiley and Sons).

The annealing process for perovskite films has the potential to lead to the volatilization of organic cations and halides, resulting in residual defects such as uncoordinated Pb^2+^, halide vacancies, and Pb clusters on the surface [104,105,106]. These positively charged defects can be viewed as Lewis acids, which can be passivated by the introduction of Lewis bases that can provide a pair of non-bonded electrons leading to the formation of Lewis acid–base adducts [15]. In initial studies, Noel et al. found that pyridine and thiophene could be used as Lewis base passivators. Due to the strong electronegativity of N and S atoms in pyridine and thiophene, efficient coordination bonds were formed between N or S atoms and uncoordinated Pb^2+^ (Figure 7a), reducing non-radiative recombination in perovskites [107]. Further research showed that derivatives of pyridine and thiophene Lewis bases were found to have further improved the passivation effects on perovskite. Cai et al. studied the passivation effects of pyridine isomers with different basicities [108]. The results showed that by pre-protonating 4-aminopyridine (4A) to modulate its proton behavior, uncoordinated organic cation defects were more effectively passivated by pyridine in perovskites, with the corresponding device yielding a PCE of 23.3%. Thiadiazole has a similar structure to thiophene. Zhu et al. added a thiadiazole derivative (1,3,4-thiadiazole-2,5-dithione, TDZDT) to the perovskite precursor solution, resulting in grains exceeding 800nm in size (Figure 7b), and the defect densities were reduced sevenfold due to C=S [109]. 

Lewis bases containing amine (e.g., -NH_2_) or nitrogen functional groups also led to effective passivation of perovskite films [110,112]. Gu et al. found that uncoordinated Pb^2+^ and Pb clusters and other defects in perovskites were all passivated by adding histamine (HA) to the surfaces of all-inorganic perovskites because surface iodine vacancies strongly bound to HA, benefitting from the additional hydrogen bonds in HA [110]. Moreover, the band edge of perovskite moved upward in the presence of HA, which promoted interface hole transport (Figure 7c), and, as a result, the highest PCE of 20.8% was achieved. Kamarudin et al. reported the role of ethylamine in tin-based perovskite solar cells, showing that the amino group in ethylamine could bind to under-coordinated tin bonds, passivating dangling bonds and defects [112]. Additionally, ethylamine can inhibit the oxidation of Sn^2+^ to Sn^4+^ and reduce recombination reactions in perovskite films. Kim et al. reported on the impact of iodinated melamine on the photovoltaic performance of perovskite solar cells [113]. The PCEs of the devices were improved and hysteresis effects were reduced due to the cyclic -C=N and primary amine in iodinated melamine. S-donor-containing and N-donor-containing Lewis base molecules function similarly to passivate halide vacancy defects. Wang et al. introduced thiourea (TU) into the perovskite precursor solution to form the thiourea intermediate phase MAI·PbI_2_·DMSO, optimizing the crystallization and morphology of perovskite films (Figure 7d) [111]. Ko further demonstrated that hydrogen bonds had formed between thiourea and perovskite or Lewis acid–base adducts, which could slow down perovskite crystal growth [114]. Meanwhile, iodine ion migration was inhibited due to the transformation of iodine molecules into I^−^ ions being reduced by thiourea, leading to the improvement of the stability of perovskite films. 

**Figure 8:**
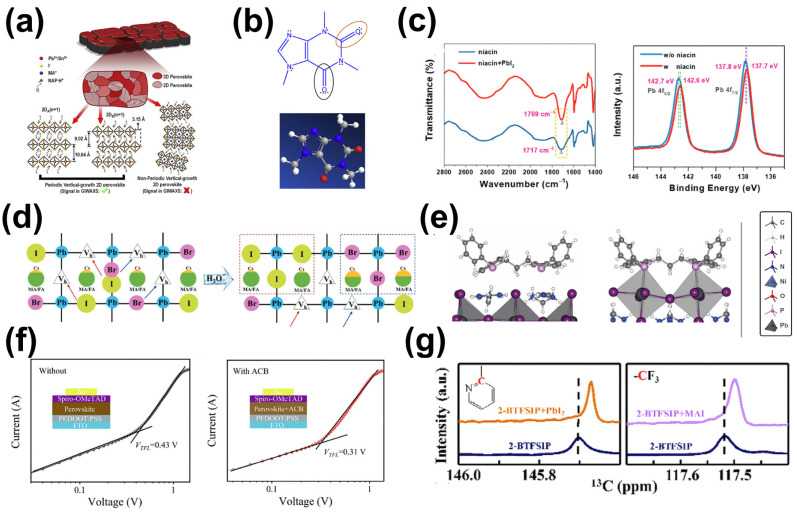
Passivation by Lewis acid. (**a**) Schematic structure of grain boundaries of 2D/3D hybridized perovskite passivation. This material was reprinted with permission from Ref. [115] (copyright 2018, Elsevier). (**b**) Lewis chemical structure and 3D structure of 1,3,7-trimethylxanthine (caffeine). This material was reprinted with permission from Ref. [69] (copyright 2019, Elsevier). (**c**) FTIR spectra of niacin and the niacin + PbI_2_ complex (**left**), and X-ray photoelectron spectroscopy (XPS) spectra for perovskite film with and without niacin (**right**). This material was reprinted with permission from Ref. [117] (copyright 2021, Royal Society of Chemistry). (**d**) Schematic representation of Cs and halide ion co-migration in the presence of halide vacancies. This material was reprinted with permission from Ref. [119] (copyright 2020, John Wiley and Sons). (**e**) Covalent and van der Waals bonding of DPPP with FAI and PbI_2_ terminally bound to the FAPbI_3_ surface calculated via DFT. This material was reprinted with permission from Ref. [105] (copyright 2023, The American Association for the Advancement of Science). (**f**) Dark I–V curves of hole-only devices that were not and were subjected ACB treatment. This material was reprinted with permission from Ref. [120] (copyright 2023, Royal Society of Chemistry). (**g**) ^13^C NMR spectra of 2-BTFSIP, 2-BTFSIP-PbI_2_ mixture and 2-BTFSIP-MAI mixture. This material was reprinted with permission from Ref. [121] (copyright 2023, Elsevier).

Other functional groups with lone pairs of electrons, for example, oxygen (O) or phosphorus (P), are also Lewis bases [68,69,115]. Chen et al. introduced N-(3-aminopropyl)-2-pyrrolidone containing the C=O functional group into CH_3_NH_3_Sn_0.5_Pb_0.5_I_x_Cl_3−x_ films, showing that the carbonyl group interacted with Sn^2+^/Pb^2+^ in the perovskite, forming vertically oriented two-dimensional layered perovskites seamlessly connected with 3D perovskite crystals (Figure 8a) [115]. The performance of C=O-modified tin-based perovskite solar cells remained above 70% after more than 1 month of continuous illumination in a nitrogen-filled glove box. Wang et al. introduced caffeine into the perovskite precursor solution (Figure 8b) and found that the C=O group in caffeine reacted with uncoordinated Pb^2+^, increasing the activation energy for perovskite growth and producing perovskite films with a preferred orientation [69]. Further studies showed that C=O could passivate reverse Pb defects in perovskites. On this basis, they further replaced theophylline as the passivator [68]. The study showed that the C=O in theobromine interacted with Pb-I reverse defects, and the N-H adjacent to the C=O could form hydrogen bonds with I in PbI_6_^2−^, enhancing the passivation effect of C=O. Therefore, perovskite solar cells prepared using theobromine additives achieved a PCE of up to 23.48%. Guan et al. demonstrated the feasibility of non-volatile carbonyl (COOH) passivation, wherein COOH-containing trimesic acid (TA) can coordinate with Pb at grain boundaries to reduce the complexation of carriers at grain boundaries, thus increasing the PCE of the device from 12.52% to 14.51% [116]. Ren et al. added 3-pyridinecarboxylic acid (nicotinic acid) with COOH to MAPbI_3_, for which the interaction of -COOH and Pb^2+^ in nicotinic acid with Pb^2+^ resulted in passivation within the perovskite (Figure 8c), as verified experimentally and through theoretical calculations [117]. As a result, the devices that contained nicotinic acid yielded a PCE close to 20%. Wang et al. introduced 3M molecules, containing both C=O and COOH, into perovskite films and demonstrated that uncoordinated Pb^2+^ defects in films were effectively passivated, achieving a maximum PCE of 24.07% [118]. 

Additives containing a P=O group are also effective at passivating electron-poor defects in perovskites [119,122]. For example, the use of triphenylphosphine oxide (TPPO), containing three benzene rings connected to the P=O double bond, has been widely reported for the passivation of uncoordinated Pb^2+^ on the surfaces of perovskites by forming P=O–Pb bonds [123]. Additionally, benzene rings with a π electron in TPPO can promote charge transfer between the perovskite and the hole transport layer. In addition, Yang et al. showed that halide segregation was suppressed because of incorporating tri(heptafluorophenyl)phosphine (TPFP) containing a P=O group and three connected F atoms with strong electronegativity into perovskite films (Figure 8d) [119]. Cheng et al. synthesized DPPO, with two phosphoric acid groups, and found that uncoordinated Pb^2+^ defects in perovskite films were passivated due to the presence of a P=O group [124]. Furthermore, the alignment of the upper levels of the perovskite was adjusted by introducing DPPO into devices, and carrier extraction was improved, leading to reduced non-radiative recombination at the interface. As a result, the PCE of the devices incorporating DPPO was 24.24%. Through theoretical calculations and experimental characterization, Li et al. demonstrated that 1,3-bis(diphenylphosphino)propane (DPPP) in phosphorus-containing molecules improves the quality of perovskite films via the formation of chemical bonds with the surface of the PbI_2_ terminus via P-Pb bonding (Figure 8e) [105]. After the DPPP-treated devices were stored in air at 85 °C for more than 1500 h, the devices showed a slight increase in PCE.

In addition to utilizing single-functional-group organic molecules for passivation, researchers have focused on using organic molecules containing multiple functional groups to synergistically passivate defects in perovskite films [125,126,127]. First, the effects of dual-functional-group synergy will be discussed. Choi et al. introduced hydroxyethyl acrylate (HEA), containing both C=O and O-H functional groups, into the perovskite layer as a functional additive [128]. The results showed that the O-H functional group in HEA could interact with organic cations (MABr and FAI) and thus form complexes. The crystallization of perovskite film was regulated, and the defects were passivated by HEA, improving the film’s crystallinity and grain size. Wu et al. reported the use of the organic small molecule 2-amino-5-chlorobenzophenone (ACB) to passivate surface defects in (FAPbI_3_)_0.95_(MAPbBr)_0.05_ perovskite films [120]. The C=O in ACB and the N donor in the NH_2_ functional group formed coordination bonds with uncoordinated Pb^2+^, while the -NH_2_ and -Cl groups also formed hydrogen bonds with the perovskite. Consequently, the trap density of films passivated by ACB decreased from 1.46 × 10^16^ m^−3^ to 1.05 × 10^16^ m^−3^ (Figure 8f), and the PCE of the perovskite solar cells passivated with ACB was 24.32%. Similarly, the carboxyl group in 2,6-pyridinedicarboxylic acid (2,6-PDA) also has a defect passivation effect. Tian et al. reported the use of various organic functional groups for defect passivation and crystallization control in perovskites. By introducing 2-[N,N-bis(trifluoromethanesulfonyl)amino]pyridine (2-BTFSIP) into the perovskite layer, they found that the sulfonyl and pyridine nitrogen groups synergistically passivated deep traps (Pb^0^ clusters and under-coordinated Pb^2+^ ions), while the trifluoromethyl group inhibited the evaporation of organic cations and increased the hydrophobicity of the perovskite film (Figure 8g) [121]. Moreover, larger crystal sizes were obtained due to the synergistic effect of these two groups. Moreover, there are also other organic molecules with multiple functional groups for synergistic passivation, such as EHA, a hydroxamic-acid-based material, and push–pull 4-hydroxybiphenylsubstituted NMI (4OH-NMI) [129,130].

Guided by Lewis acid–base passivation theory, researchers also designed and synthesized organic molecules containing various organic functional groups. Cho et al. synthesized COCH_3_ organic molecules containing methyl, carboxyl, and pyridine ring groups, further enhancing the passivation effect of defects, slowing down the nucleation and growth process of perovskites, and improving film quality [131]. Wu et al. designed π-conjugated Lewis base molecules, namely, 2-cyano-3-[5-[4-(diphenylamino)phenyl]-2-thienyl]acrylic acid (CDTA) [132]. They showed that the carbonyl (C=O) and cyanide (C≡N) groups could interact with the Sn-I framework, and the aromatic π-conjugated system could control the interaction between the Lewis base and the Lewis acid SnI_2_. Additionally, a π-spacer based on thiophene was used to increase the stability of the π-conjugated Lewis base. Consequently, FASnI_3_ films treated with CDTA showed no pinholes and a highly uniform morphology. Therefore, organic compounds with specific functional groups capable of combining with Lewis acids (C_60_, PCBM) can simultaneously act as Lewis acids and Lewis bases, promoting the development of high-efficiency and highly stable perovskite solar cells [127].

### 2.3. Polymer

As defect passivation materials, organic molecules are affected by various factors, such as light, heat, and humidity, so the defects present in perovskite films cannot be completely passivated. In contrast, polymers used as surface passivants have unique advantages. Due to their large and long-range ordered molecular structure, they can effectively facilitate persistent defect passivation under operational conditions [30,133]. Next, we will introduce two strategies for defect passivation via polymers, namely, non-in situ polymerization and in situ polymerization.

**Figure 9:**
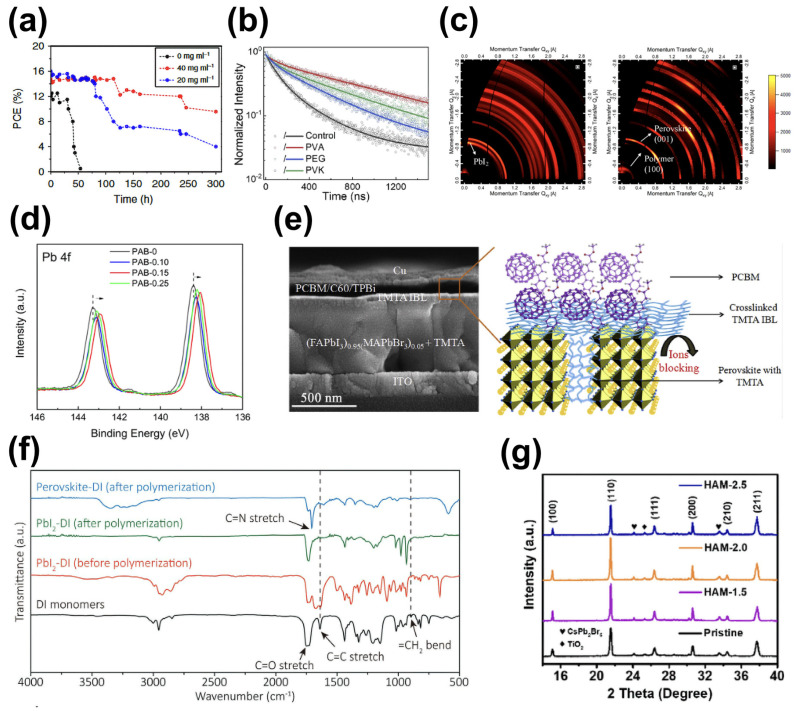
Passivation by polymer. (**a**) PCE evolution as a function of time for perovskite solar cells with different concentrations of PEG scaffold exposed to a high-humidity (70% relative humidity) dark environment without any sealing. This material was reprinted with permission from Ref. [137] (copyright 2016, Springer Nature). (**b**) Time-resolved PL decay of perovskite films with and without polymer-modified layers. This material was reprinted with permission from Ref. [138] (copyright 2022, Elsevier). (**c**) GIWAXS 2D patterns with and without PTQ10 treated during the thermal annealing process. This material was reprinted with permission from Ref. [140] (copyright 2018, American Chemical Society). (**d**) XPS spectra of Pb 4f with different amounts for PAB. This material was reprinted with permission from Ref. [142] (copyright 2022, Elsevier). (**e**) Cross-sectional SEM images of PSCs with crosslinked TMTA and schematic representation of chemically crosslinked TMTA at the titanite/PCBM interface. This material was reprinted with permission from Ref. [143] (copyright 2019, Elsevier). (**f**) Fourier transform infrared spectra (FTIR) of the DI monomers, PbI_2_-DI film before and after the polymerization process, and perovskite film after the intermolecular exchange process. This material was reprinted with permission from Ref. [144] (copyright 2020, John Wiley and Sons). (**g**) X-ray diffraction (XRD) patterns of CsPbBr_3_ films with and without HAM. This material was reprinted with permission from Ref. [145] (copyright 2022, John Wiley and Sons).

Non-in situ polymerization is a common strategy for preparing polymer-passivated perovskite solar cells. It can be carried out by mixing a polymer pure solution with a perovskite precursor solution or by introducing the polymer into the perovskite body or surface [134,135,136]. Zhao et al. significantly improved the stability of perovskite films by introducing polyethylene glycol (PEG) into a perovskite thin film through suitable temperature processes, with non-encapsulated devices maintaining high outputs for up to 300 h under conditions of high humidity (70%) (Figure 9a) [137]. Wang further compared the effects of three typical polymers, namely, polyvinyl acetate (PVA), polyethylene glycol (PEG), and poly(9-vinylcarbazole) (PVK), on the structure of perovskite [138]. They found that the surface iodide vacancy (V_I_) defects on the surfaces of the perovskite thin films were effectively passivated and the carrier diffusion ability (Figure 9b) was enhanced by PVA, exhibiting Lewis base functional groups (C=O) and low steric hindrance. Devices modified by PVA exhibited a power conversion efficiency (PCE) of 23.2%. Polymers with specific functional groups, such as poly 4-vinylpyridine (PVP), poly (methyl methacrylate) (PMMA), poly (ethylene terephthalate) (PET), poly (4-butanediyl diphenylamine) (PTPD), poly (methyl acrylate) (PMA), polyvinyl alcohol (PVA), poly(acrylic acid) (PAA), etc., can effectively passivate defects in perovskite [135,138,139]. The functional groups pyridine (C–N=C), amino (–NH_2_), carbonyl (–C=O), carboxyl (–COOH), etc., could chemically interact with ions such as Pb^2+^, I^−^, and MA^+^ in perovskite, effectively regulating the crystallization process of perovskite and improving the quality of perovskite thin films or passivating surface dangling bond defects. Meng et al. demonstrated that conjugated donor-acceptor polymers (PTQ10) triggered a reduction in the evaporation of organic cations during annealing and promoted the preferential orientation growth of perovskite crystals (Figure 9c), leading to a PCE of 21.2% [140]. Zhang et al. reported that the synthesis of amphiphilic polymer zwitterions (PMPC) with phosphocholine (PC) side chains through reversible addition-fragmentation chain transfer (RAFT) polymerization, combining various functional groups (zwitterions, Lewis bases, and carboxylic acids) in one polymer chain, could be used as a multifunctional polymer passivant [141]. PO_4_^3−^ in PMPC passivated the uncoordinated Pb^2+^ defects through P=O bonding, and, in addition, the quaternary ammonium passivated the organic cationic vacancies. At the same time, the passivator could increase the built-in potential of the device and reduce the loss of open-circuit voltage. Li et al. prepared phenol hydroxyl-substituted polyamide derivatives (PAB) containing hydroxyl, secondary amine, and carboxyl functional chain segments that increased the PCE of the studied devices from 19.45% to 21.13% due to the fact that the hydroxyl and carboxyl groups can act as Lewis bases to passivate the under-coordinated lead defects, and the secondary amine can be coordinated with iodide ions (Figure 9d) [142].

In situ polymerization means introducing polymer monomers into a perovskite precursor solution, and polymerization occurs during or after perovskite thin-film crystallization [143,144,146]. Li et al. incorporated cross-linkable organic small molecules, i.e., trimethylolpropane triacrylate (TMTA) molecules, into the perovskite layer and found that TMTA spontaneously formed a cross-connected network, which wrapped around perovskite grains and coordinated with surface dangling lead ions during the annealing of perovskite (Figure 9e) [143]. Meanwhile, the carbonyl groups (C=O) carried by TMTA chemically interacted with lead ions on the surfaces of the perovskite crystals, anchoring them at grain boundaries. As a result, the perovskite thin films were more resistant to heat, water, and light. Subsequently, Li et al. utilized a polymerizable additive, ethyl 2-cyanoacrylate (E2CA), whose CN and C=O groups coordinated with PbI_2_, to passivate perovskite grain boundaries. Moreover, a hydrophobic polymer was formed, which made the perovskite films more resistant to humid environments [147]. Zhao et al. incorporated diiodomethyl isophthalate (DI) monomers into the PbI_2_ precursor solution, in which polymerization occurred during annealing, with the polymer to grain boundaries, leading to the effective passivation of the uncoordinated lead ions (Figure 9f) [144]. Ultimately, an optimal PCE of 23% was achieved. In addition, incorporating conductive polymers into perovskite yielded better results. Gu et al. added 5-bromo-2,3-dihydrothieno [3,4-b] [1,4] dioxin (BEDOT) into a perovskite precursor solution, demonstrating that the conductive polymer poly (3,4-ethylenedioxythiophene) (PEDOT) formed through polymerization, which was helpful for the anchored Pb^2+^ on the perovskite surface and grain boundaries due to the S and O atoms contained therein [148]. Jiao et al. reported a two-step in situ polymerization strategy for preparing CsPbBr_3_ films. They introduced N-(hydroxymethyl)acrylamide (HAM) monomers containing multiple functional groups (C=C, C=O, and –NH) into the CsBr precursor solution and found that C=O····Pb(Cs) Lewis acid–base coordination had occurred and that N-H····Br hydrogen bonds at grain boundaries had formed, which were beneficial for passivating defects and delaying crystallization to control band structure (Figure 9g), effectively suppressing non-radiative recombination [145]. Unsaturated olefinic molecules are more prone to polymerization, leading to the formation polymers, with acrylic monomers being common. Monomers such as dimethylaminoethyl methacrylate (DMAEMA) and N-methylacrylamide (NMA) can undergo polymerization reactions triggered by light, heat, humidity, etc., and thus adjust perovskite grain size or passivate defects at grain boundaries, thereby yielding high-quality films for preparing high-performance perovskite solar cells [149,150].

## 3. Passivation of Defects on the Surface of Perovskite

Most perovskite solar cells have a sandwich structure, with the perovskite absorption layer situated between the electron transport layer and the hole transport layer. Interfaces between perovskite films and transport layers provide opportunities for defects to form, which are not conducive to the performance and stability of solar cells. Ni et al. used drive-level capacitance profiling to show that the defect density at the grain boundaries of polycrystalline perovskite surfaces is one to two orders of magnitude higher than the bulk defect density [31]. The defects located at interfaces will act as recombination centers, which will cause the quenching of the photogenerated carriers, leading to serious non-radiative recombination losses. As a result, the V_oc_ and FF will decrease, and the degradation of perovskite solar cells will accelerate. Therefore, reducing the defect density at the interface is also one of the crucial ways in which to further improve the PCE and stability of these devices [151,152].

### 3.1. Electron/Perovskite Transport Layer Interface

Metal oxides such as TiO_2_ and SnO_2_ are common electron transport layer materials in convention structure devices, but they have an abundance of defect sites, such as oxygen vacancies, unsaturated metal atoms, cationic interstitials, hydroxyl groups, etc., on their surfaces. These defects can cause significant V_oc_ losses and instability issues [153,154]. Moreover, The morphology and properties of the electron transport layer/perovskite layer interface (buried interface) also affect the growth of perovskite film, which, in turn, affects device performance [155]. In order to modify these defects, several materials are usually chosen, such as amphoteric ion molecules, small organic molecules, fullerene and its derivatives, metal compounds, and so on [154,156,157,158].

**Figure 10:**
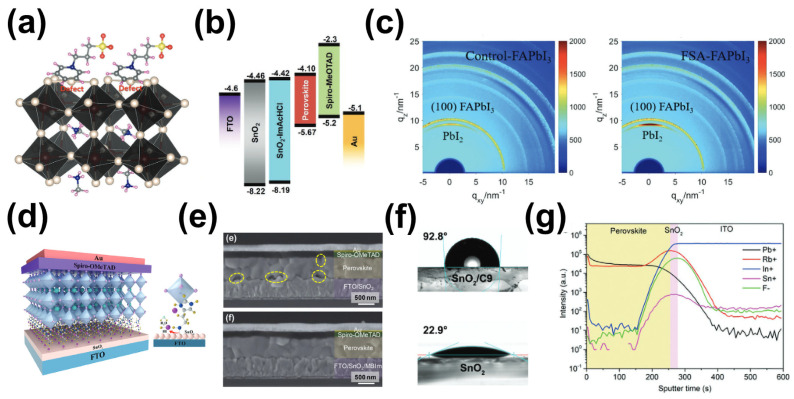
Passivation of electron transport layer/perovskite interface. (**a**) Schematic model showing passivation of Pb-I defects. This material was reprinted with permission from Ref. [160] (copyright 2018, Royal Society of Chemistry). (**b**) Energy-level diagram of devices with and without ImAcHCI. This material was reprinted with permission from Ref. [161] (Copyright 2019, John Wiley and Sons). (**c**) 2D GIWAXS patterns of the control and FSA-FAPbI_3_ perovskite films. This material was reprinted with permission from Ref. [164] (copyright 2022, John Wiley and Sons). (**d**) Schematic representation of HADI passivated defects at the SnO_2_/perovskite interface. This material was reprinted with permission from Ref. [157] (copyright 2022, John Wiley and Sons). (**e**) Cross-sectional SEM images of devices based on pristine SnO_2_ ETL and MBIm-modified SnO_2_ ETL. Holes and defects at the perovskite interface are indicated by yellow dot circles. This material was reprinted with permission from Ref. [154] (copyright 2020, Elsevier). (**f**) Contact angles of DMF on C9-modified SnO_2_ and bare SnO_2_ substrates. This material was reprinted with permission from Ref. [153] (copyright 2018, Royal Society of Chemistry). (**g**) TOF-SIMS profiles of the perovskite films deposited on ITO/SnO_2_/RbF. This material was reprinted with permission from Ref. [165] (copyright 2021, John Wiley and Sons).

Amphiphilic ionic molecules refer to materials possessing both cationic and anionic groups within the same molecule. With their participation, interface dipoles will be formed, which are conducive to charge extraction and injection. Moreover, due to the presence of both cationic and anionic moieties, amphiphilic ion molecules can establish chemical interactions with the perovskite layer and/or ETL, thereby passivating interface defects [159]. Choi et al. introduced 3-(1-pyridin-1-yl)-1-propanesulfonate as a modifier at the perovskite/ETL interface, and the addition of this modifier improved the coverage of the SnO_2_ layer, thereby promoting the growth of perovskite crystals [160]. Additionally, the modified ETL exhibited interface dipole characteristics, which suppressed charge recombination and enhanced charge transfer. Furthermore, Pb-I antisite defects at the interface were passivated by the positive charge carried by this molecule (Figure 10a), reducing the defect density on the perovskite film’s surface. Ultimately, the PSCs achieved an optimal PCE of 21.43%. Chen et al. added the ionic liquid 4-imidazolium acetate (ImAcHCI) to the buried interface, where the carboxylic acid groups in ImAcHCI formed chemical bonds with hydroxyl groups on the surface of SnO_2_, while the imidazolium cation interacted with iodide ions in the perovskite [161]. In this dual role, the energy-level alignment was optimized (Figure 10b), and instances of non-radiative recombination were reduced in the perovskite film. The research conducted by Jung et al. involved the application of ammonium fluoride (NH_4_F), a dual-functional ionic molecule, to modify the surface of SnO_2_ [162]. This process led to the modification of the surface of SnO_2_ and a reduction in defects in the SnO_2_ film through chemical doping, concurrently adjusting the energy level to optimize its properties. Separately, Zhang et al. applied potassium tetrafluoroborate (KBF_4_) to the interface between SnO_2_ and perovskite. The high-electronegativity BF_4_^−^ in KBF_4_ could reduce the hydroxyl (-OH) defects on the surface of SnO_2_, while the K^+^ ions could diffuse to the grain boundaries of the perovskite to interact with the halogen ions [163], thereby contributing to the reduction in interfacial carrier complexation, with a 22.9% PCE obtained for the KBF_4_-modified device. Qin et al. documented the modification of SnO_2_ in perovskite solar cells (PSCs) using formamidine sulfinic acid (FSA). Grazing incidence X-ray studies suggested that FSA not only reduced the work function of SnO_2_ through the generation of surface dipoles but also effectively manipulated the preferential orientation of PbI_2_ crystals (Figure 10c) [164]. Cross-sectional SEM images revealed that FSA surface treatment diminished residual PbI_2_ and hindered the development of interface defects in perovskite films; consequently, the PSCs incorporating FSA modifications achieved a PCE exceeding 24%.

Organic small molecules are also good passivators in the presence of functional groups, effectively reducing the defects density at the interface and decreasing the non-radiative recombination of charge carriers. Yang et al. employed histamine diiodate (HADI) to tailor SnO_2_, achieving a PCE of 24.79% [157]. Both experimental and theoretical research suggested that the amino terminal of HADI bound to Sn^4+^ ions within SnO_2_, while the imidazole terminal’s nitrogen atom interacted with lead atoms on the surface of the perovskite film (Figure 10d), facilitating a robust interfacial bond. This bond not only raised the conduction band edge of SnO_2_ but also mitigated lead cluster defects on the perovskite surface, which, in turn, lessened the recombination of photo-generated charge carriers at the interface. Additionally, small molecules can also exert influence over the growth dynamics of perovskite films. Sonmezoglu et al. explored the modification of SnO_2_ with 2-methylbenzimidazole (MBIm). The introduction of this molecule did not affect the grain size of the perovskite, but it significantly enhanced the film’s adherence to the SnO_2_ surface [154]. Furthermore, the nitrogen atom in MBIm coordinated with lead atoms in the perovskite, which diminishes the defect density at the interface (Figure 10e). This reduction in defect density further mitigated electron recombination at the buried interface. The interface thus passivated exhibited an improved open-circuit voltage of 1.15V for the devices. 

Fullerenes and their derivatives are widely utilized structures as electron transport layers in p-i-n. Additionally, they also serve as excellent interface modifiers for ETL/perovskite layers, facilitating charge transfer at the interface, diminishing defect recombination, and enhancing device stability [166]. Wu et al. utilized [6,6]-phenyl C_61_-butyric acid (PCBA) as a buffer layer at the TiO_2_/perovskite interface. The chemical interaction between the C_60_ end of PCBA and the electron-rich iodide in the chalcocite crystals helped to release the interfacial stresses and reduce the interfacial defects [151]. Liu et al. introduced a fullerene derivative ((9-(1-(6-(3,5-bis(hydroxymethyl)phenoxy)-1-hexyl)-1H-1,2,3-triazol-4-yl)-1-nonyl [60] fullerenoacetate (C9)) onto the surface of a SnO_2_ film, where the hydroxyl terminus in C9 can effectively form Lewis adducts with uncoordinated Sn, thus passivating the oxygen vacancy defects on the surface of the electron layer and reducing the complexation of photogenerated charges on the SnO_2_ surface. Consequently, the hydrophobicity of the SnO_2_ film’s surface was improved (Figure 10f), and the device’s efficiency was boosted to 21.3% [153]. Li et al. introduced an independently synthesized fullerene derivative, PCBB-2CN-2C8, into the TiO_2_/perovskite interface, thereby passivating the oxygen vacancies on the surface of TiO_2_, promoting the efficient transport of photogenerated carriers, and improving the stability of the device under ultraviolet light [167].

Additionally, to regulate the interface between ETL and the perovskite layer, considerable research has been conducted on the modification of interfaces using metal compounds. Studies have shown that metal halides can passivate anionic or cationic defects at the buried interface. So far, several metal halides, such as CsBr, CsCl, KCl, NaCl, KI, etc., have been used to improve device performance. Zhuang et al. introduced RbF into the buried interface and found that F^−^ interacted with surface oxygen vacancies of SnO_2_, while Rb atoms interacted with exposed oxygen atoms on the SnO_2_ surface [165]. Furthermore, Rb^+^ could migrate to the interstitial positions of the perovskite lattice (Figure 10g), inhibiting ion migration in the perovskite and reducing non-radiative recombination. Through surface treatment, devices modified with RbF achieved an excellent PCE of 23.4%, displaying a high V_oc_ of 1.21V. Huang et al. prepared water-soluble two-dimensional TiS_2_ materials and deposited them on the surface of SnO_2_, which increased the device’s PCE from 18.46% to 21.23% [168]. This improvement in device performance was attributed to the lower transmission barrier resulting from the reduced work function of the modified electron transport layer, as well as the interaction between Pb and S atoms, which can passivate defects on the perovskite surface. Moreover, metal oxides have become an ideal choice for ETL due to their appropriate energy level structure and excellent carrier mobility. What is exciting is that some metal oxides can also serve as modifying layers for ETL/perovskite interfaces. For example, MgO, Al_2_O_3_, ZrO_2_, In_2_O_3_, and SnO_2_ have all been used to suppress carrier recombination at the buried interface [169,170]. Tsvetkov et al. spin-coated PbO crystals on the buried interface and performed an analysis according to density of states [171]. The results showed that the transmission and injection of interface carriers were enhanced after introducing PbO at the interface, which has a low bandgap.

### 3.2. Perovskite/Hole Transport Layer Interface

The interface between the hole transport layer and the perovskite layer is also a major location of defects. Moisture and oxygen atoms are prone to captured by defects at the conventional structural interface, leading to the degradation of perovskite materials and reducing the long-term stability of devices. Moreover, a high defect density at the interface will lead to increased carrier recombination and altered energy level alignment, which, in turn diminish carrier transport, cause a hysteresis effect, and are detrimental to device efficiency. Therefore, reducing the defect density at the perovskite/HTL interface is also crucial for preparing PSCs with high performance [18,172]. Currently, the most prevalent research materials for defect passivation encompass organic small molecules, polymers, and substances that form low-dimensional perovskite structures [140,173,174,175,176].

**Figure 11:**
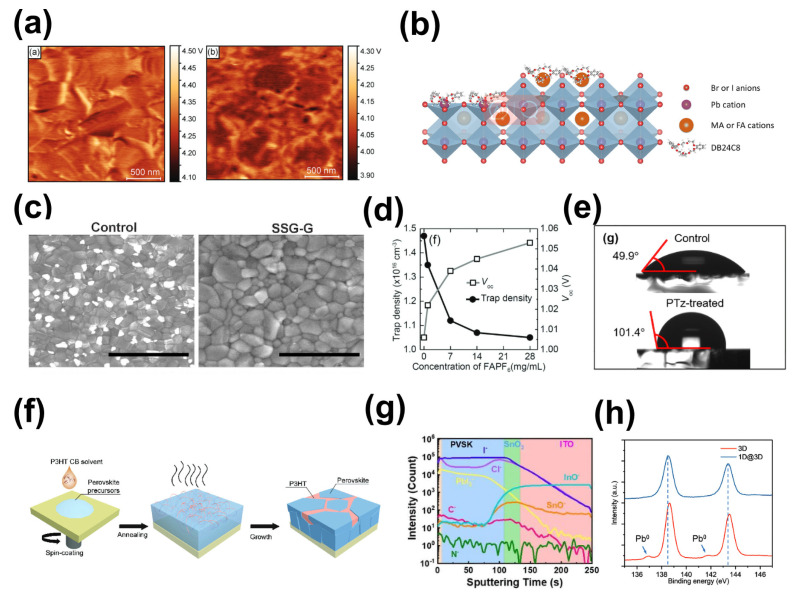
Passivation of perovskite/hole transport layer interface. (**a**) Calibrated work function map of control samples and target samples measured in ultra-high vacuum (UHV) using Kelvin probe force microscopy measurements (KPFM). This material was reprinted with permission from Ref. [173] (copyright 2021, Royal Society of Chemistry). (**b**) Schematic illustration of defects associated with crown ether modulation passivation. This material was reprinted with permission from Ref. [177] (copyright 2020, American Chemical Society). (**c**) Top-view SEM images of control and guanidine-bromide-treated films (SSG-G). This material was reprinted with permission from Ref. [178] (copyright 2018, The American Association for the Advancement of Science). (**d**) V_oc_ and trap density as a function of FAPF_6_ concentration. This material was reprinted with permission from Ref. [179] (copyright 2018, John Wiley and Sons). (**e**) Water contact angle measurement of perovskite films with/without PTz treatment. This material was reprinted with permission from Ref. [180] (copyright 2021, American Chemical Society). (**f**) Schematic illustration of P3HT/PVK heterojunction preparation. This material was reprinted with permission from Ref. [181] (copyright 2023, John Wiley and Sons). (**g**) ToF-SIMS profile of the ITO/SnO_2_/Perovskite/TBHCl. This material was reprinted with permission from Ref. [182] (copyright 2022, American Chemical Society). (**h**) Pb 4f XPS spectra of 3D and 1D@3D perovskite. This material was reprinted with permission from Ref. [176] (copyright 2022, John Wiley and Sons).

Krishna et al. introduced thiophene dicarboxylic acid into the interface. X-ray photoelectron spectroscopy and first-principles calculations revealed that the C=O group in this molecule could coordinate with an uncoordinated Pb cluster on the surface of the perovskite, thereby reducing charge accumulation at the interface [173]. Additionally, KPFM demonstrated that perovskite films modified with thiophene dicarboxylic acid exhibited an upward band bending trend at grain boundaries (Figure 11a), helping to reduce the interface energy barrier and accelerate carrier injection at the interface. Consequently, the corresponding n-i-p PSCs achieved a PCE exceeding 23%. Sutanto et al. synthesized small molecules based on phosphine oxide to modify the perovskite/HTL interface, and a PCE of 20.7% was achieved due to the coordination between oxygen atoms in the molecule and lead atomic defects in the perovskite film [174]. Su et al. proposed complexing crown ether molecules with cations in perovskite, with the aim of passivating surface defects [177]. Their results demonstrated that the cationic atoms in crown ether molecules could effectively interact with uncoordinated Pb^2+^, MA^+^, and FA^+^ cations in the perovskite film (Figure 11b). This interaction reduced surface defect states, thereby minimizing losses in open-circuit voltage. The devices treated with crown ether molecules achieved a PCE of 23.7%. Furthermore, the incorporation of crown ether molecules not only decreased the reactivation of defect sites by water and oxygen within the film but also improved the film’s hydrophobicity, with both affordances being beneficial to improving the stability of the devices. Organic molecules can also engage in secondary reactions with excess PbI_2_ on the surface, which will enhance interface quality. For example, Luo et al. reduced the excess PbI_2_ on the surface of perovskite by introducing guanidine bromide (GABr) on the surface of the perovskite film to allow it to react with PbI_2_ (Figure 11c), which reduced the Pb-associated defects on the surface of the perovskite and thus enhanced device stability [178]. Chen et al. treated the surface of FA_0.88_Cs_0.12_PbI_3_ with formamidine hexafluorophosphate (FAPF_6_), facilitating a surface solid-state ion exchange that led to the formation of a perovskite phase containing PF_6_^−^ anions [179]. This new phase acted as grain boundary patches, significantly reducing the trap density to 1.05 × 10^16^cm^−3^ (Figure 11d). Owing to the hydrophobic nature of fluorine, the resulting device demonstrated enhanced stability against water and oxygen. 

The polymer modified at the perovskite/HTL interface not only improves a device’s V_oc_ by suppressing defect-induced compounding but also provides improved device stability. Li et al. synthesized a naphthalene diimide (NDI)-based conjugated polymer bearing 3,4-difluorothiophene (PTzNDI-2FT) and applied it at the interface between the perovskite layer and the Spiro-OMeTAD layer [180]. Oxygen and fluorine atoms in the PTzNDI-2FT were provoked to interact with the uncoordinated Pb elements on the perovskite surface, in turn suppressing the defective states and increasing the diffusion length of the carriers. In addition, elemental fluorine enhanced the hydrophobicity of the perovskite film surface (Figure 11e). As a result, the prepared devices achieved a PCE of 23.2%. Kim et al. incorporated the multifunctional hygroscopic polymer polyethylene oxide (PEO) on the interface of perovskite/HTL, showing that PEO not only provided electrons to under-coordinated lead ions to reduce defects but also prevented water adsorption on the perovskite surface [183]. However, the majority of polymer-based passivation interfaces employ insulating polymers, and the low conductivity of these materials could detract from device performance. Consequently, researchers have sought to develop conjugated polymers that exhibit semiconductor properties and are amenable to film deposition. Li et al. dissolved p-type or n-type conjugated polymers in the antisolvent chlorobenzene and subsequently spin-coated them on the surface of perovskite. The morphology of the perovskite films was optimized, and defects were passivated without affecting the grain size of the perovskite [184]. Yang et al. applied the common conjugated polymer P3HT to the surface of the perovskite layer through spin-induced line decomposition (a spontaneous solid-phase separation process), forming an interpenetrating P3HT/perovskite heterojunction (Figure 11f) [181]. As a result, the energy-level alignment was adjusted, and interface defects were passivated, which separately reduced energy loss and carrier quenching at the interface, achieving an efficiency of 24.53%. Akman et al. applied poly (N,N’-bis-4-butylphenyl-N,N-biphenyl)benzidine (polyTPD), a derivative of triphenylamine, to the perovskite/HTL interface. polyTPD passivated the defects at the surface and grain boundaries by penetrating into the inner layer of perovskite, a process that was achieved using a passivation strategy wherein a central nitrogen atom in polyTPD interacted with the Pb^2+^ defects [185]. The specific passivation process is as follows: the central nitrogen atom in polyTPD forms a Lewis adduct with the uncoordinated Pb^2+^ defects, inhibiting the formation of Pb^0^ defects.

The introduction of a low-dimensional chalcogenide layer at the upper interface is enabled by utilizing the interfacial passivation reaction between an organic amine salt and the perovskite surface. In 2018, Chen et al. spin-coated 5-aminovaleric acid hydroiodide (5-AVAI) onto a perovskite surface [186]. Upon reaction with the excess PbI_2_ present on the surface, 5-AVAI facilitated the formation of a 2D perovskite layer at the perovskite/CuSCN interface, denoted as (5-AVAI)_2_PbI_4_. This strategic interface engineering successfully passivated defects, thereby mitigating hysteresis effects in the device. Liu et al. introduced tert-Butylcarbamidine hydrochloride (TBHCI) onto the surface of a perovskite film to prepare gradient three-dimensional and two-dimensional heterojunction perovskite films [182]. Time-of-flight secondary ion mass spectrometry (ToF-SIMS) showed that TBHCI exhibited a gradient distribution in the perovskite film (Figure 11g), thereby controlling the gradient distribution of the valence band of the perovskite film and enhancing carrier transport. Finally, PSCs with an efficiency of 22.54% were prepared. Sung et al. studied the effect of [2-(9H-carbazol-9-yl)ethyl] phosphonic acid (CEPA) on the band structure of perovskite films. The results showed that CEPA raised the valence band of the perovskite by 0.1 eV, improving the band matching of the perovskite film and the transport layer, which helped to enhance carrier transport at the interface. Ultimately, the PCE of the modified device was 23.6% [187]. In addition, lower-dimensional organic cations were also used for passivating defects on the surface of three-dimensional perovskite films. For example, Wu et al. applied thiopheniformamidine hydrochloride based on the structure of thiophene to the surfaces of perovskite films. The cations of thiopheniformamidine hydrochloride reacted with PbI_2_ and MAI, yielding one-dimensional perovskite crystals, resulting in the formation of one-dimensional and three-dimensional heterojunctions (1D@3D) [176]. Simultaneously, the sulfur in the thiopheniformamidine hydrochloride was effective in passivating Pb clusters in the perovskite film (Figure 11h), which, in turn, enhanced the photogenerated carrier lifetime of the film. Chen et al. applied 1,10-phenanthroline (Phen), a bidentate ligand with two lone pairs of electrons, to the perovskite surface, where the ion in Phen can reacted with the excess PbI_2_ on the surface to form a 1D PbI_2_ (Phen) adduct phase as a passivation layer, and, at the same time, the N atoms in the Phen interaction can reacted with uncoordinated Pb^2+^ on the surface of the perovskite, which can suppress carrier complexation at the interface on perovskite, based on which the PSCs obtained were has a PCE of over 23% [175].

## 4. Conclusions and Outlook

Currently, defect passivation in perovskite is an essential and effective strategy for enhancing the photovoltaic performance and stability of perovskite solar cells as well reducing hysteresis phenomena. This review systematically summarizes the current common passivators used for passivating defects in perovskite films, which mainly include ionic compounds, organic ammonium salts, Lewis acid–base molecules, and polymers. In general, passivators improve device performance mainly through the following ways: (1) chemical reaction with defects inside or on the surface of perovskite crystals; (2) optimizing the nucleation and crystallization process of perovskite films, assisting in forming dense, uniform, high-quality perovskite films; (3) adjusting the energy level alignment between perovskite and the charge transport layer to promote effective charge extraction; and (4) improving device stability by preventing external moisture infiltration or suppressing ion migration. Due to the above functions, passivation has emerged as an indispensable optimization strategy for improving the efficiency and stability of PSCs.

Although defect passivation techniques are becoming increasingly sophisticated, there remains a lack of a profound understanding of the underlying passivation mechanisms. Additionally, the precise characterization of defect types, concentrations, and the depths of defects is challenging, which precludes the selection of the most appropriate passivating agents for defect mitigation. At the same time, it is impossible to accurately select effective passivating agents using existing theories. Therefore, in the future, improvements in the photovoltaic performance of devices can be made in relation to the following aspects: (1) Synergistic passivation strategies. These involve developing more-efficient passivating agents and integrating various complementary functions to achieve a synergistic passivation effect, thereby enabling multifunctional molecules to more effectively modify perovskite films. (2) Atomic-scale analysis strategies. The accurate identification and mapping of defect types are crucial for the regulation of defects, and they can be achieved through the use of high-resolution aberration-corrected transmission electron microscopy, in situ X-ray diffraction (XRD), and other advanced characterization techniques. These methods allow for in situ analysis of perovskite films, enabling the resolution of defects at the atomic scale and providing a more profound understanding of the passivation mechanisms. (3) Machine learning. Machine learning can be used to discover the basic principles behind selecting passivating materials and optimizing devices.

In conclusion, understanding the methods and mechanisms of defect passivation is very beneficial to the further improvement of the efficiency of batteries. This article systematically introduces the defects in the perovskite layer and its passivation mechanism, laying the foundation for further searches for effective passivation materials to improve the performance of the perovskite solar cells.
